# Advances in Immunotherapy for Glioblastoma Multiforme

**DOI:** 10.1155/2017/3597613

**Published:** 2017-02-19

**Authors:** Boyuan Huang, Hongbo Zhang, Lijuan Gu, Bainxin Ye, Zhihong Jian, Creed Stary, Xiaoxing Xiong

**Affiliations:** ^1^Department of Neurosurgery, Beijing Electric Hospital, State Grid, Beijing 100073, China; ^2^Department of Neurosurgery, Hubei Provincial Hospital of Integrated Chinese and Western Medicine, Wuhan, Hubei 430015, China; ^3^Central Laboratory, Renmin Hospital of Wuhan University, Wuhan, Hubei 430060, China; ^4^Department of Hematology, Renmin Hospital of Wuhan University, Wuhan, Hubei 430060, China; ^5^Department of Neurosurgery, Renmin Hospital of Wuhan University, Wuhan, Hubei 430060, China; ^6^Department of Anesthesiology, Perioperative and Pain Medicine, Stanford University School of Medicine, Stanford, CA 94305, USA

## Abstract

Glioblastoma multiforme (GBM) is the most common primary malignant brain tumor in adults. Patients with GBM have poor outcomes, even with the current gold-standard first-line treatment: maximal safe resection combined with radiotherapy and temozolomide chemotherapy. Accumulating evidence suggests that advances in antigen-specific cancer vaccines and immune checkpoint blockade in other advanced tumors may provide an appealing promise for immunotherapy in glioma. The future of therapy for GBM will likely incorporate a combinatorial, personalized approach, including current conventional treatments, active immunotherapeutics, plus agents targeting immunosuppressive checkpoints.

## 1. Introduction

Glioblastoma multiforme (GBM) is the most common primary malignant brain tumor in adults, accounting for approximately 60–70% of gliomas [1] and 15% of primary brain tumors [2]. The current standard treatment for patients with GBM is maximal tumor resection followed by adjuvant radiotherapy and temozolomide [3]. Although this standardized treatment has demonstrated efficacy in prolonging patient survival, the prognosis for patients remains extremely poor, with a median survival time (MS) of 14.6 months and an average 5-year survival rate of less than 5% [1, 2, 4]. This may be partly due to resistance of GBM cells to treatment and their capacity to spread and invade into surrounding brain parenchyma. Accordingly, substantial efforts have been made in developing new approaches for gene therapy, targeted chemotherapeutics, and/or radiotherapeutic modalities. However, the MS for patients with newly diagnosed GBM have improved only modestly during the past 10 years.

Immunotherapy, harnessing the power of the host's immune system by inducing, enhancing, or suppressing immune responses to reject cancer cells, is rapidly becoming a pillar of anticancer therapy. Immunotherapeutic approaches can be classified as active immunotherapy aimed at promoting a T_h_1 immune response through tumor vaccines, nonspecific immune stimulants, or cellular vaccines, and passive immunotherapy, to induce an antitumor effect by transferring effector immune cells into patients. In 2010, the first antigen-specific vaccine for castration-resistant prostate cancer, sipuleucel-T, was approved by the FDA. In 2011, the first checkpoint inhibitor for advanced melanoma, ipilimumab, was also approved. Since then, immunotherapy has proven effective in the treatment of melanoma, Hodgkin's lymphoma, renal cell carcinoma, and non-small-cell lung cancer (NSCLC) in which conventional therapies have gained limited success [5–9] (Table 1). In this review, we will summarize the application of immunotherapy for GBM and discuss preclinical data and emerging clinical studies of vaccination, immune checkpoint blockade, and adoptive T-cell transfer in the treatment of this devastating disease.

## 2. CNS Immune Privilege and Immunosuppression of GBM

The central nervous system (CNS) has been traditionally viewed as an immune-privileged site, secondary to the blood-brain barrier (BBB) that prevents free diffusion of cells and molecules and lack of a conventional lymphatic drainage system [10–13]. Paradoxically, however, it has been known for over 20 years that brain tumors have the capacity to elicit potent antitumor immune responses. Most recently, the discovery of a CNS lymphatic system has provided an explanation for this phenomenon. Using animal bearing intracranial tumors models, it has been demonstrated that tumor-derived antigens can be drained from the cerebrospinal fluid into the cervical lymph nodes to stimulate specific T-cells [14]. After amplification, these T-cells are able to efficiently migrate into the CNS and target and kill tumor cells [15]. However, these so-called tumor-specific T-cells have to exert their function in a hypoxic environment, where chronic inflammation and tumor cells can stimulate immunosuppression [16]. In addition, the inflammatory stimuli introduced by brain tumors can induce microglial activation and blood-brain barrier (BBB) disruption. Microglia serve as the main effector cells of the innate immune system in the CNS and play a critical role in cytotoxicity against phagocytosis and T-cell activation through antigen presentation. It has been demonstrated that microglia can increase GBM cell migration and invasion via secretion of matrix-degrading enzymes and membrane type I metalloproteinases (MMPs) [17]. The role that microglia plays in GBM tumor progression was verified by the identification of protumorigenic Osteoactivin (GPNMB) and Osteopontin (SP1) expression in profiled GBM tumor-associated microglia [18]. Disruption of the BBB with injury and disease can facilitate the presentation of CNS antigens to the cervical lymph nodes, serving to prime T-cells for homing and infiltration into the tumor parenchyma [19–21].

**Figure 1:**
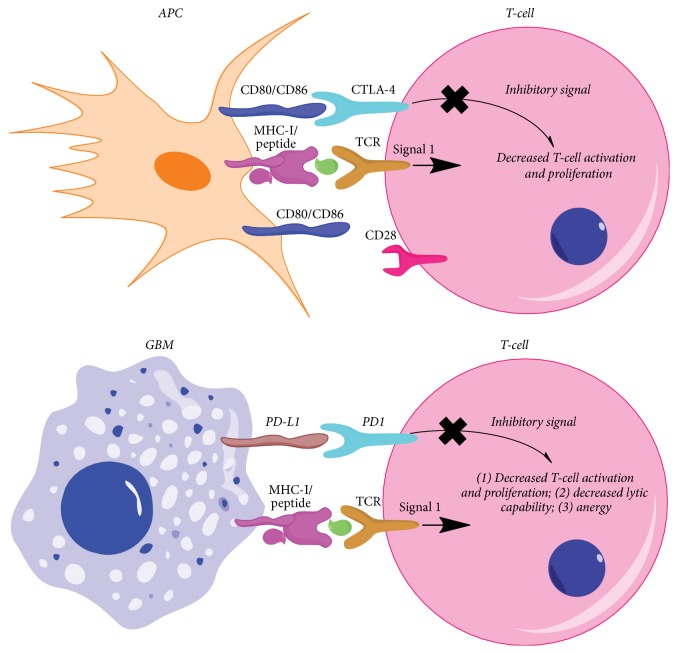
Mechanism of CTLA-4 and PD-L1 immune checkpoints. The CTLA-4 immune checkpoint (left figure) occurs early during the priming phase of the immune response, acting within secondary lymphoid organs. CTLA-4 is a powerful inhibitory T-cell receptor that can preferentially bind to CD80/CD86 on the surface of APCs, preventing their binding to the T-cell costimulatory receptor CD28, thus leading to decreased T-cell activation and proliferation in the context of antigen-presenting MHC class I. PD-1 signaling takes place during the effector phase of the immune responses within the tumor microenvironment. The inhibitory PD-1 T-cell receptor interacts with one of two currently identified PD-1 ligands: PD-L1 or PD-L2, expressed on the surface of tumor cells. Engagement of PD-1 ligands with the PD-1, in the context of tumor antigen-presenting MHC class I, can decrease the T-cell tumor lytic capacity and induces T-cell anergy. APC: antigen-presenting cell.

In GBM, a high level of vascular endothelial growth factor (VEGF) expression and pathologically structured microvessels can introduce increased permeability of BBB, enhancing the interaction between tumor cells and the immune system. GBM cells express high levels of MHC and Fas which play a role in the adaptive immune response. However, GBM has been traditionally considered an immunosuppressive tumor, effective in evading the immune response through a variety of mechanisms (Figure 1). First, GBM can express various potent immunosuppressive factors, such as indoleamine 2,3-dioxygenase (IDO), TGF-*β*, and STAT3 [22–24]. IDO is expressed in 96% of resected GBM, of which the upregulation is correlating with a poor patient prognosis [25]. IDO1 functions to convert tryptophan into kynurenines, which mediate apoptosis of effector T-cells and activation of regulatory T-cells- (Treg-) mediated immunosuppression [26]. Inhibition of TGF-*β*/Smads signaling can restore immune surveillance in glioma models [27] which could inhibit proliferation through microRNA-182 and platelet-derived growth factor-*β* (PDGF-*β*). Second, another immunosuppressive pathway mediated by interactions between programmed death 1 (PD-1) and programmed death-ligand 1 (PD-L1) contributes to the inhibition of T-cell activation and proliferation. Examination of 135 GBM specimens demonstrated that PD-L1 was positively expressed in 88% newly diagnosed GBM patients and 72% recurrent GBM patients [28]. Although the PD-L1 expression in the healthy CNS parenchyma surrounding GBM is very low, GBM cells express a relatively higher level of PD-L1 than other tumors (~30% of melanomas [29] and 25–36% of NSCLC [30]). Moreover, both tumor-infiltrating macrophages and microglia in GBM were reported to express high levels of PD-L1, suggesting the need for optimal immunotherapeutic benefit [31, 32]. A third predominant and essential pathway contributing to immunosuppression in GBM is mediation by cytotoxic T-lymphocyte antigen-4 (CTLA-4), a coinhibitory receptor that outcompetes costimulatory receptor, CD28, for binding to CD80 and CD86 [33, 34]. The inhibitory effects of CTLA-4 occur largely in naive and resting T-cells and act to inhibit T-cell effector function and augment the inhibitory activity of Tregs [35].

## 3. T-Cell Based Vaccine Therapies

Recent expansion in our knowledge of immune-mediated mechanisms has led to the rapid development of immune-targeted therapeutic strategies (Table 2). Among anticancer immunotherapies, the success of tumor vaccines and T-cell therapies relies on the elicitation of significant numbers of tumor-specific T-cells to seek and destroy tumor cells. Adaptation of vaccination strategies in cancer is aiming at eliciting unproductive immune responses against tumor cells in the patient by injection of tumor-derived antigens. The primary requirement for a safe and effective tumor vaccine is that the antigen target be expressed specifically in tumor cells but absent in normal cells of the body. In this regard, tumor-specific antigens (TSAs) that arise from mutations in the tumor are ideal candidates. An example of a potential TSA in GBM is epidermal growth factor receptor variant III (EGFRvIII), which induces the immune system to act against the tumor by presenting the mutant peptide to the stimulated immune cells [36]. EGFRvIII is the result of an in-frame deletion of exons 2–7 on EGFR resulting in a novel amino acid sequence and a truncated protein with an altered extracellular domain epitope [37]. Phase II clinical trials of Rindopepimut™, a 13-amino acid EGFRvIII peptide vaccine conjugated to adjuvant has demonstrated vaccine immunogenicity and increased overall survival (OS), which is correlated with the magnitude of induced tumor immunity [38–40]. Interestingly, most patients that relapsed after vaccination had lost the EGFRvIII antigen, demonstrating at the same time the efficacy of vaccine-induced immune responses in eradicating tumor cells [38]. Another phase III ACT IV study involved 700 patients with newly diagnosed EGFRvIII-positive GBM demonstrated that treatment of Rindopepimut (Rintega) plus temozolomide failed to improve overall survival (OS) compared with temozolomide and a control [41]. However, as reported in a study using single-cell DNA analysis, only a subset of cells in the tumor may express EGFRvIII due to the intratumoral heterogeneity, and expression may be highly variable [42, 43], resulting in survival and recurrence of the non-EGFRvIII-expressing cells. Despite these concerns, trials of Rindopepimut have shown promising results overall, leading to an ongoing phase III trial in newly diagnosed (NCT01480479) and relapsed (NCT01498328) GBM. Unfortunately, since EGFRvIII is only present in 20–30% of newly diagnosed GBM [44], the identification of alternative GBM TSAs with higher levels of expression will likely be necessary to achieve higher efficacy. For example, another clinical trial based on the mutant isocitrate dehydrogenase type 1 (IDH1) for recurrent grade II astrocytoma (NCT02193347) has shown greater efficacy [45]; the mutant IDH1 is carried by more than 70% of diffuse grade II and III gliomas [46].

Considering that heterogeneity of TSAs in the patient population as a potentially limiting factor in treatment efficacy, tumor-associated antigens (TAAs), which are not tumor exclusive but are relatively overexpressed compared to normal tissues, may be a more viable target in tumor vaccines. Clinical trials in GBM patients, using peptide-pulsed dendritic cells or peptides alone in adjuvant, demonstrated that TAA-based vaccine could elicit T-cell responses without collateral autoimmunity, showing benefit in some patients [47–50]. Early results were exciting, prompting initiation of more clinical trials, such as applying the vaccine in patients with lower-grade glioma, oligodendroglioma, oligoastrocytoma, and ependymoma (NCT01795313). On the other hand, peptide elution from GBM cells was demonstrated capable of identifying 10 novel GBM-associated antigens, brevican, chitinase 3-like 2, Chondroitin sulphate proteoglycan, fatty acid-binding protein 7, insulin-like growth factor 2 messenger RNA-binding protein 3, neuroligin 4, X-linked, neuronal cell adhesion molecule, protein tyrosine phosphatase, receptor-type, Z polypeptide, tenascin C, were overexpressed in 80–100% of GBM patients, making a peptide vaccine possible [51]. In this study, researchers found >6000 HLA-bound peptides from HLA-A^*∗*^02^+^ glioblastoma, of which over 3000 were restricted by HLA-A^*∗*^02. They prioritized investigation of these 10 glioblastoma-associated antigens, to which GBM patients showed no T-cell tolerance. Moreover, researchers demonstrated that these 10 peptides were highly immunogenic not only in healthy individuals but also in GBM patients, 9 of which were being developed in a multipeptide therapeutic vaccine designated IMA950. Moreover, peptide elution from GBM cells identified 10 novel GBM-associated antigens which are overexpressed in 80–100% of GBM patients, making a peptide vaccine a potential reality [51]. Three trials that incorporate these well-characterized TAAs (called the IMA950 antigens) are underway (NCT01403285, NCT01920191, NCT01222221), using CD8^+^ T-cell epitopes with different adjuvants. Other trials aiming at eliciting both CD4 and CD8 T-cell responses use whole proteins as immunogen to construct the TAA vaccines (NCT00626483, NCT01522820, NCT00390299).

Vaccines that target single antigens are restricted to the relatively small subset of patients with tumors that express those TSAs and TAAs. Moreover, the heterogeneity of tumor cells in expressing such antigens may also potentially limit the utility and efficacy of these single-antigen vaccines. Accordingly, alternative vaccine approaches have been created to target a broad range of antigens. Among these, heat-shock protein (HSP) peptide complexes (HSPPC-96) have generated particular interest. HSPPC-96 is a primary resident chaperone of the endoplasmic reticulum and binds various client proteins that are involved in the antigen-presenting pathway [52]. When conjugating to tumor peptides, intracellular and extracellular HSPs coordinate to mediate the internalization of HSPPC-96 into APCs for efficient class I and II MHC-mediated presentation of tumor peptides [53]. Thus, HSPPC-96-tumor peptide complexes can generate potent tumor-specific immune responses. In a phase II trial for surgically resectable recurrent GBM, in which HSPPC-96-loaded antigens were extracted from patient-derived glioma tissue to use as a personalized antiglioma vaccine, the median OS was increased to an impressive 42.6 weeks, a substantial survival benefit when compared to historical controls [54]. However, immunotherapeutic approaches may be complicated by immunogenic side effects profiles, for example, HSPPC-96 stimulation of both cytotoxic T lymphocytes (CTLs) and Tregs, especially at higher doses [55], and lymphopenia [54].

## 4. Alternative Immune-Mediated Vaccines

The concept of vaccine immunotherapy involves priming antigen-presenting cells (APCs) with tumor-derived antigens in order to accelerate the eradication of tumor cells [56] (Figure 1). Of the three types of professional APCs, dendritic cells (DCs) are the most powerful and efficient in activating T-cells, making DCs attractive candidates for therapeutic antitumor strategies [57]. DCs express high levels of cell surface markers MHC class I, MHC class II, and CD86 [58] and are involved in both innate and adaptive immune systems [59]. Compared to other APCs, DCs process antigens more slowly generating a longer and more sustained T-cell response [60]. Autologous DCs exposed to GBM-associated antigens to take up and process the antigens as peptides on their cell surface in the context of MHCs are injected back into patients as a vaccine therapy. Not only can the T-cells of patients be activated by DCs-based vaccines via recognition of MHC class I or II molecule, but natural killer (NK) and natural killer T (NKT) cell function can be improved, both of which can also elicit a powerful antitumor effect [61]. The efficacy of DC-based vaccine for GBM utilizing pulsed autologous DCs with tumor lysate is currently tested in a phase III trial for newly diagnosed GBM patients (NCT00045968). A preclinical study demonstrated that modulation of CMV-specific DCs with tetanus/diphtheria (Td) preconditioning could increase DC migration to vaccine site-draining lymph nodes (VDLNs) [62]. This DC migration could also be enhanced by exogenous administration of chemokine CCL3 in a mouse model with normal CD4 T-dependent immune responses. The investigators propose CCL3 as a novel and important mediator to increase DC migration to VDLNs. In this study, researchers found that Td-treatment could not only increase the PSF and OF in GBM patients but also suppress the tumor growth in their established mouse model. Accordingly, strategies aiming at modulating the DC migration may be a promising therapeutic option. However, the modification of autologous DCs is an expensive, time-consuming, and labor-intensive process that must be carried out in specialized facilities. In addition, the variability of some antigens in inducing immune responses may also result in variable and inconsistent effects. Tumor-specific proteins and peptides that represent these proteins have then been used as antigens to enhance tumor-specific cytotoxicity [63].

Another approach uses an immunotherapeutic strategy to target glioma stem cells (GSCs). With their more active DNA repair mechanisms and highly expressed multidrug resistance genes, GSCs may play a role in mediating the resistance of GBM to radiotherapy and chemotherapy and contribute to local immunosuppression in the GBM microenvironment [64–66]. Several studies have demonstrated that GSC-antigens-loaded DC vaccines could induce immune-reactivity and a survival benefit in rodent orthotopic GBM models [67, 68]. Another study showed that immunization with GLAST peptides, a neural stem cell marker that is highly expressed in the plasma membrane of GSCs, could efficiently prevent the tumor progression in a glioma GL261 mouse model [69]. Clinically, a DC vaccine (ICT-107) loaded with six synthetically processed GBM-associated peptides, four of which (HER2, TRP-2, AIM-2, and IL13R*α*2) are considered GSC-associated, has shown promising results in phase II trial for newly diagnosed GBM patients [70]. Another phase I trial found that median PFS and OS in newly diagnosed GBM patients were 16.9 and 38.4 months, which were correlated with expression of the GSCs associated antigens in tumors before vaccination [47]. Accordingly, GSC-antigens, however, may be ideal for vaccination for their capability of stimulating T-cells to induce tumor-specific cytotoxicity against GBM cells when loaded to DCs [71].

Several studies have demonstrated that GSC-antigens-loaded DC vaccines could induce immune-reactivity and a survival benefit in rodent orthotopic GBM models [69, 70]. Clinically, a DC vaccine (ICT-107) loaded with six synthetically processed GSC-associated peptides has shown promising results in phase II trials for newly diagnosed GBM patients [47]. Accordingly, GSC-antigens, however, may be ideal for vaccination for their capability of stimulating T-cells to induce tumor-specific cytotoxicity against GBM cells when loaded to DCs [71].

## 5. Immune Checkpoint Inhibition

It has been recognized that coinhibitory receptors on T-cells play an essential role in attenuating the strength and duration of T-cell-mediated immune responses. These inhibitory receptors are referred to as immune checkpoint molecules responsible for maintaining self-tolerance and preventing autoimmune reactions [72, 73]. To date, the two most intensely investigated coinhibitory molecules are CTLA-4 (that acts early in T-cell activation) and PD-1 (that blocks T-cells at later stages of the immune response) [74]. It has been demonstrated that blockade of CTLA4 and PD1 could induce tumor regression and promote long-term survival in mouse glioma models (Table 3) [35, 75]. Clinically, ipilimumab, a humanized CTLA-4 antibody and the first FDA-approved immune checkpoint inhibitor, has been demonstrated to improve OS in a phase III clinical trial for metastatic melanoma patients [76], however, with only a complete response observed in 2% patients. In phase I and II trials of solid tumors, ipilimumab improved PFS [77, 78] but with severe immune adverse effects [79]. However, another CTLA-4 antibody, tremelimumab, failed to show significant survival benefit in phase III trial for metastatic melanoma patients [74]. In GBM, robust antitumor immunity introduced by CTLA-4 mAb was only observed in at the preclinical stage [75] and the clinical utility of ipilimumab may be limited to only a small subset of GBM patients.

Conversely, efforts aimed at inhibiting the PD-1/PDL1 pathway have shown more promising results. In a preclinical study using the GL261 glioma mouse model, combination of anti-PD-1 antibodies and radiotherapy doubled median survival and elicited long-term survival in 15–40% of mice compared with either treatment alone [75]. Clinically, pembrolizumab, a PD-L1 antibody, has been approved by the FDA to apply in the treatment of metastatic melanoma and NSCLC. In GBM, nivolumab, another PD-1 antibody, developed for GBM patients is being tested, with two clinical trials currently recruiting GBM patients (NCT02337491, NCT02336165). The most promising results have been achieved in a randomized control trial with combinatorial CTLA-4/PD-(L)1 blockade for advanced melanoma, in which combination of CTLA-4 and PD-1 blockade demonstrated an improved objective response rate (ORR) of 58%, compared to monotherapy of anti-CTLA-4 (19%) and monotherapy of anti-PD-1 (44%) [80]. A randomized phase III study aimed at testing nivolumab versus bevacizumab in recurrent GBM patients will also test combination therapy of nivolumab and ipilimumab (NCT02017717). Another two phase I/II trials will analyze the effectiveness of combinatorial pembrolizumab with bevacizumab (NCT02337491) and combinatorial pembrolizumab with MRI-guided laser ablation (NCT02311582) in recurrent GBM patients. In addition, MEDI4736, a humanized PD-Ll mAb, is currently being tested in clinical trials for GBM patients combined with radiotherapy and bevacizumab (NCT02336165).

However, relatively high frequency of immune-related adverse effects, such as endocrinological, hepatic, gastrointestinal, and dermatological toxicities, have limited enthusiasm for immune checkpoint blockade as a immunotherapeutic strategy against cancer [81]. These adverse effects were considered to be associated with aberrant infiltration of stimulated CD4^+^ and CD8^+^ T-cells into normal tissues in company with elevated levels of proinflammatory cytokines [82]. Recently, newer agents targeting PD-1 ligands (PD-lLs) have now been tested in renal cell cancer, NSCLC, and melanoma (NCT00729664). These agents have shown the capability of inducing durable tumor regression with less grade 3 or 4 adverse events compared with CTLA-4 mAb and PD-1 mAb [83]. Overall, the combination of various immune checkpoint modulators have shown promising effectiveness in the treatment of some solid tumors. The application of combinatorial checkpoint modulators in GBM and other tumors therefore requires further investigation into the interplay of costimulatory and coinhibitory molecules.

## 6. Adoptive T-Cell Therapy

While previously described therapeutic strategies endeavored to induce endogenous T-cell responses,* adoptive* T-cell therapies provide an alternative strategy: in vitro amplification of tumor-specific autologous T-cells followed by venous infusion into the same individual. Adoptive T-cell therapy has evolved during the past two decades in concert with the development of genetic engineering, resulting in the generation of high avidity tumor-specific T-cells. Tumor-reactive T-cells are often achieved by transducing the patient's autologous T-cells with vectors encoding T-cell receptors (TCR) or chimeric antibody receptors (CAR) [84]. Although TCR engineering has not yet been applied in glioma, several preclinical studies of CARs targeting proteins (IL-13 receptor [85, 86], Her2 [87, 88], EphA2 [89], and EGFRvIII [90, 91]) have shown promising results. Clinically, adoptive T-cell therapy has demonstrated its effectiveness with CAR-based treatment for CD19C B-cell malignancies [92]. A clinical trial for 11 recurrent GBM patients has demonstrated infusions of autologous adoptively transferred human cytomegalovirus- (CMV-) specific T-cells increased OS to of >57 weeks, with 4 patients maintaining no progression throughout the study period [93]. Another clinical trial concerning CMV adoptive T-cell therapy is ongoing (NCT00693095). The next step of adoptive T-cell therapy for GBM patients will likely involve transducing autologous T-cells with CAR. CAR, which consist of the antigen-binding region of a monoclonal antibody fused with a T-cell cytoplasmic signaling domain, acts independently of MHC I expression on tumors [94]. Clinical trials investigating CAR targeting EGFRvIII (NCT02209376, NCT01454596), HER2 (NCT01109095), and IL-13R*α*2 (NCT02208362) are underway, and therapeutic benefits without unacceptable toxicity are anticipated.

## 7. Conclusions

Current open clinical trials of immunotherapy predominantly focusing on DC vaccines and antibodies targeting immunosuppressive checkpoints have achieved promising immune activity and clinical responses (see Tables 1 and 2 for summary). However, durable and sustained responses remain rare, highlighting the need for novel promising approaches including gene therapy and combinatorial immunotherapeutic treatment. Immunogenic side effect profiles underlie the need for next-generation immunotherapies with nonimmunosuppressive and/or anti-inflammatory approaches. Current obstacles for immune therapy for GBM lie in (1) finding drugs to penetrate the BBB; (2) identifying specific, suitable, and immunogenic tumor antigens; and (3) identifying appropriate pre- and posttherapeutic biomarkers. Other challenges include the limited number of GBM patients eligible to join particular clinical studies and a deep understanding of various regulatory and stimulatory factors in the immune system and GBM microenvironment. Considering that the brain tumors will ultimately metastasize outside the CNS, one future direction of immunotherapy is to design immunotherapies to obtain sufficient functional antitumor T-cell in the CNS, with no other sites to be targeted. If so, one challenge will be determining tolerable levels of inflammation to occur without damage to the brain. Additionally, there remains a need for standardized and validated assays to measure the immune response. However, increased efforts have been dedicated to establishing reliable biomarkers to improve the assessment of clinical efficacy to guide therapeutic decision-making [95]. Immune therapy for GBM requires an integrated effort, with combinations of vaccines, cell therapy, and molecules targeting the tumor environment, trying as well to exploit the beneficial aspects of radio- and chemotherapy. This will serve to improve and promote the development of an optimal personalized therapeutic strategy for the treatment of GBM.

## Figures and Tables

**Table 1:** Stage of clinical development of immunotherapeutics in select cancers.

Cancer type	Mechanism	Agent	Phase
Melanoma	Anti-CTLA-4	Ipilimumab	FDA approved
Melanoma	Anti-PD-1	Nivolumab	Phase III
Melanoma	Anti-PD-1	Pembrolizumab	FDA approved
Melanoma	Adoptive cell therapy		Phases I-II
Melanoma	Peptide Vaccine	Melan-A peptides	Phases I-II
Melanoma	Autologous DC vaccine		Phases I-II
Melanoma	Whole tumor cell vaccine		Phases I-II

NSCLC	Peptide vaccine	EGFR peptide	Phase III
NSCLC	Anti-CTLA-4	Ipilimumab	Phase III
NSCLC	Anti-PD-1	Nivolumab	Phase II

**Table 2:** Representative clinical trials of vaccine therapy in glioma.

Registration number	New/recurrent/metastatic	Therapy	Number of patients	Phase
*EGFRvIII vaccine*				
NCT01480479	New	Rindopepimut/GM-CSF	*n* = 700	Phase III
NCT00626015	New	EGFRvIII peptide vaccine, daclizumab	3 experimental versus 3 control	Pilot
[96]	New	DC vaccine targeting EGFRvIII antigen	*n* = 12	Phase I
[38]	New	EGFRvIII peptide vaccine	*n* = 18	Phase II
[39]	New	EGFRvIII peptide Vaccine, TMZ	*n* = 22	Phase II
[40]	New	Rindopepimut (CDX-110)	*n* = 65	Phase II

*Heat-shock protein (HSP) vaccine*				
NCT01814813	Recurrent	HSPPC-96 C, bevacizumab	*n* = 222	Phase II
[54]	Recurrent	HSPPC-96 vaccine	*n* = 41	Phase II
[97]	New	HSP70 vaccine	*n* = 12	Pilot

*Dendritic cell (DC) vaccines*				
NCT00846456	New	DC vaccine against cancer stem cells	*n* = 11	Pilot
NCT00068510	New + recurrent	C vaccine, toll-like receptor agonists	*n* = 23	Phase I
NCT00045968	New	DCVax®-L	*n* = 300	Phase III
[98]	New	DC vaccine	*n* = 10	Pilot
[99]	New	DC vaccine	*n* = 8	Pilot
[100]	New	DC vaccine	*n* = 5	Pilot
[101]	Recurrent	DC vaccine	*n* = 9	Phase I
[47]	New + recurrent	multi-epitope pulsed DC vaccine	*n* = 21	Phase I
[102]	New + recurrent	DC vaccine	*n* = 17	Phase I/II

*Adoptive T-cell therapy*				
NCT02209376	New + recurrent	CAR T-cells to EGFRvIII	*n* = 12	Phase I
NCT00693095	New	CMV-autologous lymphocyte transfer	*n* = 12	Phase I
NCT01109095	Recurrent	CMV-specific cytotoxic T lymphocytes	*n* = 16	Phase I
NCT01454596	Recurrent	CAR T-cells to EGFRvIII	*n* = 160	Phase I/II
NCT02208362	Recurrent + refractory	Enriched T-cells expressing IL13Ra2	*n* = 44	Phase I
[93]	Recurrent	CMV-specific T-cells	*n* = 19	Phase I

**Table 3:** Representative clinical trials of immune checkpoint blockade in glioma.

Registration number	New/recurrent/metastatic	Mechanism	Therapy	Number of patients	Phase
NCT02017717	Recurrent	Anti-PD1, anti-CTLA4	Nivolumab, ipilimumab, bevacizumab	*n* = 440	Phase III
NCT02336165	New + recurrent	Anti-PDL1	MEDI4736, Bevacizumab,	*n* = 84	Phase II
NCT02311920	New + recurrent	Anti-PD1, anti-CTLA4	TMZ, nivolumab, ipilimumab	*n* = 42	Phase I
NCT02337491	Recurrent	Anti-PD1	Pembrolizumab, bevacizumab	*n* = 79	Phase II
NCT01952769	Recurrent	Anti-PD1	Pidilizumab	*n* = 30	Phase I/II
